# Approach motivation and loneliness: Individual differences and parasympathetic activity

**DOI:** 10.1111/psyp.14036

**Published:** 2022-03-01

**Authors:** Karen E. Smith, Seth D. Pollak

**Affiliations:** ^1^ Department of Psychology University of Wisconsin – Madison Madison Wisconsin USA

**Keywords:** approach, decision making, heart rate variability, loneliness, motivation, parasympathetic nervous system

## Abstract

Loneliness, or perceived social isolation, is linked to a number of negative long‐term effects on both mental and physical health. However, how an individual responds to feeling lonely may influence their risk for later negative health outcomes. Here, we sought to clarify what influences variability in individuals' motivated responses to loneliness. Specifically, we assessed whether resting parasympathetic activity, a physiological marker linked to flexible adaptation, facilitates increased approach‐oriented behaviors. Seventy‐four adult participants underwent a conditioning paradigm assessing how they approach and avoid rewards and threats. Individuals with higher levels of loneliness and high resting parasympathetic activity were more likely to demonstrate approach behaviors. We discuss these findings in terms of the role resting parasympathetic activity may play in facilitating adaptive responses to feeling socially isolated.

## Abstract 2

How an individual responds to feeling lonely may influence their risk for later negative health outcomes. The current study provides new insight into what may influence variability in responses to feeling lonely. Individuals with higher resting parasympathetic activity and high levels of loneliness demonstrate increased approach‐oriented behaviors. This suggests loneliness increases approach motivations but only in the presence of other markers of adaptive responding, like high resting parasympathetic activity.

## 1 INTRODUCTION

Loneliness, also referred to as perceived social isolation, can have long‐lasting effects on both mental and physical health. Extended experiences of loneliness are linked to increased risk for depression, poorer general health, altered immune functioning, and increased mortality, among numerous other effects (Hawkley & Capitanio, 2015; Norman et al., 2011). However, how individuals respond to and cope with loneliness can impact their long‐term outcomes (Hawkley et al., 2012; Park & Baumeister, 2015; Vanhalst et al., 2018). In particular, approach‐oriented responses such as seeking social connections may alleviate feelings of loneliness while avoidance‐oriented responses may exacerbate them, thereby altering individuals' risk for later negative outcomes. In the current study, we aimed to elucidate what influences variability in individuals' approach‐ and avoidance‐oriented responses to loneliness. Specifically, we examined whether parasympathetic nervous system regulation, previously identified as a marker of more flexible and adaptive regulation, facilitates approach‐oriented responses to feeling lonely.

Loneliness is defined as the distressing feelings associated with perceiving oneself to lack sufficient social connections to meet one's social needs (Qualter et al., 2015; Smith & Pollak, 2021b). For social species, having strong social connections plays a critical role in facilitating survival—allowing for collaboration and cooperation that aids in obtaining resources, like food and shelter, and avoiding potential threats (Decety et al., 2012; Ochsner, 2019; Taylor, 2011). Given the reliance of social species on others, loneliness is thought to represent a salient motivational drive. It signals a potential threat to survival and has been hypothesized to facilitate motivated behaviors aimed at addressing that threat—like the maintenance and repair of social relationships (Cacioppo et al., 2011; Qualter et al., 2015; Smith & Pollak, 2021a). In support of this, there is some evidence suggesting loneliness or social exclusion increases social approach behaviors. Experimental social exclusion is associated with greater interest in making new friends and working with others, as well as more positive affect during social inclusion and loneliness has been linked to increased sensitivity to social reward (Inagaki et al., 2016; Maner et al., 2007; Van Roekel et al., 2014). However, other evidence suggests that loneliness increases withdrawal and avoidance, with reported loneliness being linked to increased negative affect during social interactions, decreased motivation to participate in social gatherings, and increased sensitivity to negative emotional information in faces (Preece et al., 2021; Smith et al., 2020; Vanhalst et al., 2018). These findings indicate that individuals vary in how they respond to feeling lonely, and that variability may have implications for their long‐term well‐being; for example, approach responses aimed at building social relationships may alleviate feelings of loneliness while avoidance responses may lead to withdrawal and increased feelings of social isolation.

Despite this, there is still relatively little research examining what may influence variability in loneliness‐related motivated behaviors. One potential factor that could provide insight into these differences is autonomic nervous system functioning. The autonomic nervous system (ANS) consists of two branches, the parasympathetic (PNS) and sympathetic nervous systems (SNS). Together, the PNS and SNS act to dually innervate organs throughout the body, facilitating adaptive motivated responses to potential threats and challenges in the environment (Berntson et al., 2006; Porges, 2011; Weissman & Mendes, 2021). The ANS is innervated by higher level cortical and subcortical systems that play an important role in motivated responding, including social engagement (Cacioppo et al., 2000; Kemp et al., 2017). The PNS in particular is thought to index activity in these cortical motivational circuits (Koenig, 2020; Porges, 2015; Smith et al., 2017). Because of this, PNS activity is thought to facilitate adaptive motivational behaviors. Specifically having high resting PNS activity has been linked to increased self‐regulation and emotion regulation, increased sensitivity to social information, and decreased avoidance behaviors along with increased sensitivity to cues of safety (Beauchaine, 2015; Katahira et al., 2014; Smith et al., 2020; Wendt et al., 2015). Given this, resting PNS activity may moderate how loneliness influences approach and avoidance behaviors. Specifically, individuals with high resting PNS, which is associated with more flexible and adaptive responding, may be more likely to respond to feeling lonely by increasing approach behaviors.

Here we assessed whether resting PNS activity moderates the relationship between loneliness and individual variability in approach and avoidance behaviors. Seventy‐four adult participants completed a task in which they learned relationships between neutral and valued (positive and negative) outcomes. They were then asked to use that information to approach and avoid the positive and negative outcomes. We expected loneliness to increase approach behaviors, but only in individuals with higher levels of resting PNS activity. If high resting PNS activity is associated with increased approach behaviors in lonely individuals, this suggests resting PNS may buffer individuals from some of the negative effects of loneliness through the facilitation of more adaptive behavioral responses to feeling lonely.

## 2 METHOD

### 2.1 Participants

We aimed to recruit 70 participants, consistent with prior research on similar topics (Fanning et al., 2020; Inagaki et al., 2016; Zhang & Gao, 2015) and recommendations from power simulation studies for hierarchical linear models (Kerkhoff & Nussbeck, 2019). Final recruitment was 74 adults (46 female) between the ages of 18–46 years old (*M* = 19.74; *SD* = 3.64; Race: 52.7% White Non‐Hispanic; 28.4% Asian; 1.4% Black/African American; 4.1% White Hispanic; 4.1% Hispanic; 5.4% Multi‐Racial; 4.1% Other). All participants provided written informed consent and received either course credit or a cash payment ($20) for participation. This study was approved by the University of Wisconsin‐Madison Institutional Review Board.

### 2.2 Procedure

Participants attended one laboratory session lasting approximately ninety minutes. Participants were first instructed to sit quietly for a 5‐min baseline assessment of physiological measures during which they watched a neutral video of colored balls moving across the computer screen, similar to those employed in previous research (Gilissen et al., 2007; Jones et al., 2014; Smith & Pollak, 2021c). Participants then completed a two‐part task aimed at assessing how they approach and avoid positive and negative stimuli. Tasks were presented using E‐Prime 2.0 on a touch screen Windows PC. An electrocardiogram (ECG) was collected using a standard lead II system throughout the experiment. All participants also completed the Three‐Item Loneliness Scale to measure perceptions of social isolation (Hughes et al., 2004). This scale is a modified version of the UCLA Loneliness Scale in which participants are asked to rate how they feel about different aspects of their life using a three point Likert scale (“Hardly Ever,” “Some of the Time,” “Often.”) Items include statements such as “How often do you feel you lack companionship?” and “How often do you feel left out?” In the current sample, the scale exhibited good internal consistency (*α* = .81). To control for any potential differences in cognitive functioning, the Matrix Reasoning and Vocabulary subtests of the Wechsler Abbreviated Scale of Intelligence‐Second Edition were administered to all participants (WASI‐II) (Wechsler, 2011). Post‐experiment all participants were debriefed.

### 2.3 Approach and avoidance task

**FIGURE 1:**
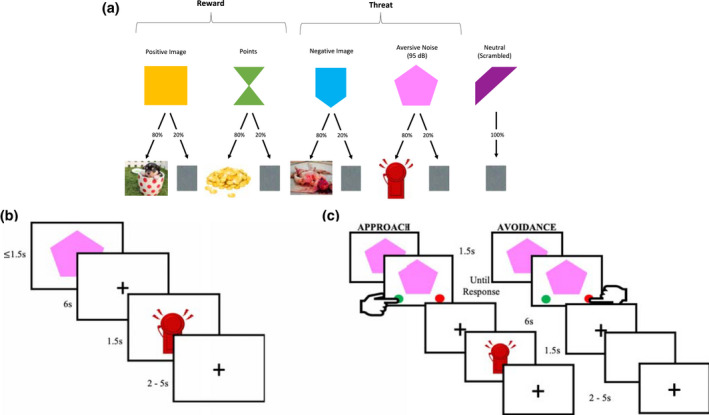
Task schematics. (a) Example of neutral shape—Reinforcer pairings and probability ratios. Neutral shapes were paired with either a positive image, points reward, negative image, or aversive noise 80% of the time and neutral scrambled image 20% of the time. One shape was always paired with the neutral scrambled image. (b) Example of a trial in the conditioning task. (c) Example of a trial in the behavioral choice task. Pressing the green button resulted in presentation of the reinforcer; pressing the red button resulted in presentation of a blank screen. Thus, pressing the green button represents an approach response and pressing the red represents an avoidance response. Figure is adapted from Smith and Pollak (2021c)

The approach avoidance task consisted of two parts. The first part of the task consisted of a Pavlovian conditioning paradigm where they saw five colored shapes followed by either appetitive, aversive, or neutral reinforcers (Metereau & Dreher, 2015). Appetitive reinforcers consisted of points and a positive image; aversive reinforcers were an unpleasant 95 dB noise and a negative image (Figure 1). The images were taken from the Open Affective Standardized Image Set (OASIS; Kurdi et al., 2017; Positive Image: I256; Negative Image: I287). During conditioning, participants saw a visual cue (geometric colored shape) that was displayed until a keyboard response was made or 1.5 s had passed. This cue was followed by a delay period of 6 s during which a fixation cross was displayed. The delay was followed by either a corresponding reinforcer or a scrambled neutral image presented for 1.5 s with a probability of 0.8 for the reinforcer and 0.2 for the scrambled neutral image. Each trial was followed by a jittered inter‐trial interval of 2.5–5.5 s. A fifth neutral condition consisted of a geometric cue always followed by the neutral scrambled picture. To maintain attention and as a measure of conditioning, participants were asked to press a keyboard response button as soon as they saw the geometric cue. Participants completed 14 trials of each condition for a total of 70 trials. Presentation of each trial was randomized within participants. Across participants, the shape‐reinforcer pairings were counterbalanced using a Latin Square design. To ensure participants learned the shape reinforcer relationships, participants were asked to rate how good or bad they thought each neutral shape was prior to and after the conditioning task using a Visual Analogue Scale. Visual Analogue Scale ratings ranged from 0 (Bad) to 100 (Good).

After the conditioning task, participants completed a behavioral choice task in which they were asked to use value information from the conditioning task to approach or avoid appetitive and aversive stimuli. This task was the same as the conditioned learning task, with the following exceptions. On each trial, participants were presented with the same shapes they had encountered on the previous task. After 1.5 s, a green and a red button appeared on either side of the screen. These buttons remained on screen until participants made a response (Figure 1). If participants selected the green button, the trial proceeded as in the conditioning task—the paired reinforcer was presented with a probability of 0.8 for the reinforcer and 0.2 for the scrambled neutral image. However, if participants selected the red button, a blank screen appeared without any reinforcer. In this manner, selecting the green button represented an approach response and selecting the red button represented an avoidance response. As in the conditioning task, participants completed 14 trials of each condition for a total of 70 trials. Trial presentation was randomized within participants and the side of the screen where the green and red buttons appeared was counterbalanced across participants.

### 2.4 Physiological measures

To assess resting PNS activity, we derived high frequency heart rate variability (HF‐HRV), a measure of parasympathetic cardiac control, from the ECG collected during the 5‐min baseline period of the study. HF‐HRV is a rhythmic fluctuation of heart rate in the respiratory frequency band (respiratory sinus arrhythmia [RSA]) and has been demonstrated to be a relatively pure index of parasympathetic cardiac control (Berntson et al., 1997). HF‐HRV was derived from ECG using spectral analysis of the IBI series. This time series was detrended (second‐order polynomial), end tapered, and submitted to a fast Fourier transformation. HF‐HRV spectral power was then integrated over the respiratory frequency band (0.12–0.40 HZ) and HF‐HRV is represented as the natural log of the heart period variance in the respiratory band (in ms^2^).

### 2.5 Statistical analyses

To assess whether participants learned the neutral shape—reinforcer pairings, we used a three‐level hierarchical linear model (HLM; lmer function in the lme4 package in R v3.5.1) with the shape ratings during conditioning as the outcome. This model included a random intercept for reinforcer type, with reinforcer type nested within participant and fixed effects for rating time (pre−/post‐conditioning) and reinforcer type (neutral, positive image, negative image, points, and aversive noise) as fixed predictors. To test the associations between loneliness, resting PNS, and participants' approach and avoidance behaviors we ran a three‐level hierarchical logistic regression (glmer function in package lme4). In this model, approach and avoidance behavior was coded as a binary outcome—with approach coded as “1” and avoid coded as “0.” Thus, outcomes are reported in terms of changes in probability of approach behaviors, but this model can be used to also make inferences about avoidance—an increase in probability of approach is indicative of a reciprocal decrease in probability of avoidance. This model included a random intercept for reinforcer type and reinforcer type nested within participant, along with reinforcer type, reported perceived social isolation, resting PNS activity, and an interaction between reinforcer type, perceived social isolation, and resting PNS activity as fixed predictors. Significance of all fixed effects was assessed using the Anova function in the car package. Interactions were examined by calculating estimated marginal effects for predicted response probabilities at different levels (*M* and ± 1 *SD*) for the continuous predictors (Long & Mustillo, 2021; McCabe et al., 2021) using package emmeans. Loneliness (*M* = 5.29, *SD* = 1.81) and resting PNS activity (*M* = 5.95, *SD* = 1.34) were not correlated (*r* = −.20, *p* = .09), suggesting any interaction effects are not driven by loneliness being associated with higher or lower resting PNS (or vice versa). We ran all analyses controlling for age and gender. As has been done previously (Hanson et al., 2017; Mukherjee et al., 2020), to ensure any effects were not driven by individual variability in general cognition, we also ran all analyses controlling for general cognitive ability. Additionally, given some research suggests effects of resting PNS activity may be accounted for by heart rate (de Geus et al., 2019), we ran the analyses controlling for resting heart rate.

## 3 RESULTS

### 3.1 Validation of conditioned learning

To ensure participants learned the neutral‐shape reinforcer pairings, we first examined changes in their Visual Analogue Scale. Participants appeared to have learned the neutral shape—reinforcer pairing, (*χ* ^2^[4] = 58.09, *p* < .001), rating shapes paired with positive reinforcers more positively after conditioning (Points: *β* = 7.15, *SE* = 2.84, *p* = .01; Positive Image: *β* = 8.86, *SE* = 2.84, *p* = .002) and shapes paired with negative reinforcers more negatively after conditioning (Aversive Noise: *β* = −15.54, *SE* = 2.84, *p* < .001; Negative Image: *β* = −11.16, *SE* = 2.84, *p* < .001).

### 3.2 Approach and avoidance behaviors

To assess whether participants approached appetitive and avoided aversive reinforcers, we examined their behavior on the behavioral choice task. Participants differed in their likelihood of approaching the different reinforcers (*χ* ^2^[4] = 327.85, *p* < .001) in the expected direction. Participants were more likely to approach positive reinforcers (Points: *M*_approach_ = 0.99, *SE* = 0.003, CI: [0.99, 1.00]; Positive Image: *M*_approach_ = 0.96, *SE* = 0.01, CI: [0.93, 0.98]) and avoid negative reinforcers (Aversive Noise: *M*_approach_ = 0.13, *SE* = 0.03, CI: [0.06, 0.19], Negative Image: *M*_approach_ = 0.20, *SE* = 0.05, CI: [0.11, 0.29]). Together this suggests participants effectively learned the relationships and used the information to inform their motivated approach and avoidance behaviors.

### 3.3 Loneliness and resting PNS in relation to approach and avoidance behaviors

**FIGURE 2:**
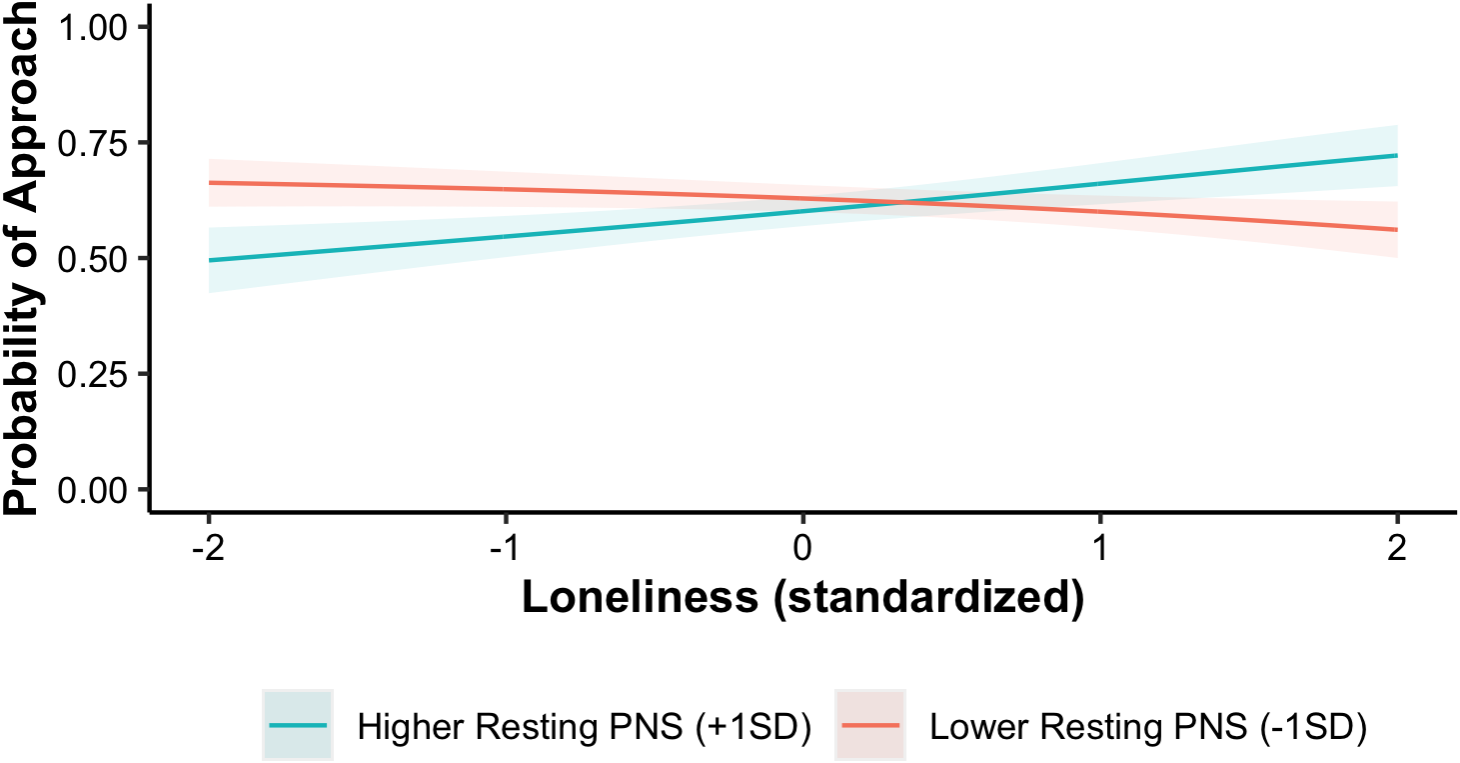
Relationship between loneliness, resting PNS, and probability of approach behaviors. Higher levels of loneliness were associated with increased approach behaviors but only in individuals with higher resting PNS

As hypothesized, there was an interaction between loneliness and resting PNS in the expected direction (*χ* ^2^[1] = 6.28, *p* = .01; Figure 2). Specifically, participants with higher resting PNS activity and higher levels of loneliness demonstrated an increased probability of approaching reinforcers (*β* = .11, *SE* = 0.06, *p* = .05). In contrast, individuals with lower resting PNS activity demonstrated little relationship between approach behaviors and loneliness (*β* = −0.05, *SE* = 0.05, *p* = .29). There were no main effects of loneliness (*χ* ^2^[1] = 0.01, *p* = .93) or resting PNS (*χ* ^2^[1] = 0.02, *p* = .90) on approach behaviors. These effects did not change when controlling for age, gender, cognitive ability, and baseline heart rate.

While not the primary question of interest, it could be that individuals high in loneliness with high resting PNS increase approach behaviors due to poorer learning during the conditioning task. To determine if this was the case, we also examined the effects of loneliness and resting PNS on changes in participants' Visual Analogue Ratings of the shapes. There were no effects of loneliness (*χ* ^2^[4] = 5.75, *p* = .22) or resting PNS activity (*χ* ^2^[4] = 2.92, *p* = .57) on changes in Visual Analogue Ratings (Interaction: *χ* ^2^[4] = 7.35, *p* = .12). Controlling for age, gender, cognitive ability, and baseline heart rate did result in the interaction between loneliness, resting PNS, reinforcer condition, and time of rating (pre/post‐conditioning) becoming significant (*χ* ^2^[4] = 11.17, *p* = .02). However, this appeared to be driven by individuals with lower resting PNS and low levels of loneliness rating the negative image more negatively post‐conditioning (*β* = −26.89, *SE* = 9.78, *p* = .03) and the points reward more positively post‐conditioning (*β* = 32.73, *SE* = 9.78, *p* = .01). There was not evidence for differences in ratings of the other reinforcer types linked to loneliness and resting PNS (*p*s > .10).

## 4 DISCUSSION

In the current study, we assessed if resting PNS activity moderates differences in individuals' loneliness‐related approach and avoidance motivations. We found that whether an individual responded to feeling lonely by increasing their propensity to approach or avoid positive and negative outcomes depended on their resting PNS activity. Individuals with high levels of loneliness and high resting PNS activity demonstrated increased approach behaviors and decreased avoidance behaviors. In contrast, individuals with low resting PNS activity demonstrated little relationship between loneliness and approach behaviors. Together this suggests that loneliness increases approach motivations but only in the presence of other markers of adaptive responding, like high resting PNS activity.

Our data add to the existing literature on loneliness and approach and avoidance motivations, illuminating one potential mechanism which may explain divergent findings. Loneliness represents a salient motivational cue—signaling a lack of strong and high‐quality social relationships. Given this, increases in approach motivations may represent an adaptive response strategy to feeling lonely, supporting the seeking of new and maintenance of existing social relationships (Cacioppo et al., 2011; Hawkley & Capitanio, 2015). Here, we find that individuals with high resting PNS activity are those who increase their approach behaviors in response to feeling lonely. Overall, these differences suggest that individuals with higher resting PNS may be better able to cope with feelings of loneliness, using them to seek out new and maintain existing relationships, placing them at less risk for extended experiences of loneliness and the associated negative outcomes. One alternative explanation for these findings is that resting PNS activity leads to increased loneliness and through that shapes approach and avoidance behaviors. However, in the current study loneliness and resting PNS were not correlated suggesting this is likely not the case. Research utilizing a longitudinal approach can better establish whether there is a causal relationship between loneliness and PNS activity and how this influences approach and avoidance motivations. It is also possible that individuals with high levels of loneliness and high resting PNS activity were less able to encode the shape—reinforcer relationships during the learning task. While we do find some evidence of potential differences in learning, they are only apparent after controlling for covariates and are specific to the negative image and points reinforcer. This specificity suggests they do not contribute to the more general changes in approach behaviors. Research examining these questions in a larger sample across a range of reinforcers can better assess how these differences in learning may contribute to changes in motivated behaviors.

The moderating effect of resting PNS activity on the relationship between loneliness and approach and avoidance behaviors is likely due to it indexing activity in prefrontal cortical neural circuits critical to facilitating flexible adaptation to the environment. Indeed, having high resting PNS activity has been associated with increased emotional and cognitive regulation (Beauchaine, 2015; Park et al., 2012) and covaries with activity in prefrontal cortical circuits during tasks that tap these regulatory processes (Smith et al., 2017; Thayer et al., 2012). Our findings are in line with this previous work—supporting a role of high PNS facilitating more flexible responding to environmental challenge; in this case feeling socially isolated. Future research can examine whether individuals with high resting PNS activity also demonstrate differential engagement of these prefrontal cortical circuits when making decisions about whether to approach or avoid stimuli.

The current research can be expanded on in future studies in several ways. We examined approach and avoidance motivations broadly, in the context of varying types of stimuli, and did not manipulate the sociality of stimuli. We find no effect of reinforcer type, suggestive of increased approach behaviors for regardless of stimulus type. This is in contrast theories that posit loneliness has a specific effect on social motivations (Cacioppo et al., 2011; Hawkley & Capitanio, 2015; Qualter et al., 2015). However, it is possible participants assigned sociality to stimuli typically considered to be non‐social (i.e., the aversive noise and points reward)—viewing them as social feedback. Research directly manipulating the sociality of stimuli along with asking participants about their perceptions of sociality can better examine whether the effects of loneliness are specific to social approach and avoidance (Bangee et al., 2014; Qualter et al., 2015).

Our study also did not allow us to compare more acute experiences of loneliness with more chronic loneliness; acute loneliness has been hypothesized to increase approach while more chronic loneliness lead to avoidance (Qualter et al., 2015; Vanhalst et al., 2018). The Three‐Item Loneliness Scale (and other questionnaire‐based measures of loneliness) are traditionally considered to be trait measures of chronic loneliness (Hughes et al., 2004; Vanhalst et al., 2018). However, there has been little research assessing changes in these measures over time. The research that has examined this question suggests there is meaningful variation in trajectories of loneliness that have implications for health and well‐being (Qualter et al., 2013; Smith et al., 2020; Vanhalst et al., 2013). Future research examining longitudinal trajectories of loneliness can provide further insight into whether acute and chronic loneliness differentially affect social approach and avoidance motivations. Last, our sample consisted of a primarily White undergraduate sample which somewhat limits the generalizability of the findings. Examining these questions in a more diverse sample can aid in better understanding whether they are comparable across different populations.

Overall, this study provides insight into one potential mechanism through which loneliness shifts approach and avoidance motivations. In particular, our findings suggest resting PNS may be one marker of risk for negative loneliness‐related outcomes that can be elaborated on in future research. They also indicate that individuals vary in how they respond to loneliness and points to a need for further research examining what factors contribute to this variation. Research examining behaviors in more naturalistic settings can aid in better understanding how shifts in motivation linked to loneliness and resting PNS activity shape social interactions. This type of research can aid in illuminating potential targets of intervention for loneliness.

## CONFLICTS OF INTEREST

The authors have no conflicts of interest to disclose.

## AUTHOR CONTRIBUTIONS

**Karen Smith:** Conceptualization; data curation; formal analysis; investigation; methodology; project administration; visualization; writing – original draft; writing – review and editing. **Seth D. Pollak:** Conceptualization; funding acquisition; investigation; project administration; resources; software; supervision; writing – review and editing.

## Data Availability

Associated data and code are publicly available on the Open Science Framework (OSF; https://osf.io/hs8ju/).
